# Targeting Cellular Senescence with Liposome-Encapsulated Fisetin: Evidence of Senomorphic Effect

**DOI:** 10.3390/ijms26157489

**Published:** 2025-08-02

**Authors:** Agata Henschke, Bartosz Grześkowiak, Olena Ivashchenko, María Celina Sánchez-Cerviño, Emerson Coy, Sergio Moya

**Affiliations:** 1 NanoBioMedical Centre, Adam Mickiewicz University, Wszechnicy Piastowskiej 3, 61-614 Poznan, Poland; agata.henschke@amu.edu.pl (A.H.);; 2 Biomedical Polymers Division, Research Institute for Materials Science and Technology (INTEMA), National University of Mar del Plata (UNMdP)-National Scientific and Technical Research Council (CONICET), Av. Colón 10850, Mar del Plata 7600, Argentina; 3 Soft Matter Nanotechnology Group, CIC biomaGUNE, Basque Research and Technology Alliance (BRTA), Paseo Miramón 182, San Sebastián, 20014 Guipúzcoa, Spain

**Keywords:** senescence, fisetin, liposomes, senotherapy

## Abstract

Cellular senescence is closely connected with cancer progression, recurrence, and metastasis. Senotherapy aims to soothe the harmful effects of senescent cells either by inducing their apoptosis (senolytic) or by suppressing the senescence-associated secretory phenotype (SASP) (senomorphic). Fisetin, a well-studied senotherapeutic drug, was selected for this study to evaluate its efficiency when delivered in a liposomal formulation. The experiment evaluated the impact of liposome-encapsulated fisetin on senescent cells induced by doxorubicin (DOX) from two cell lines: WI-38 (normal lung fibroblasts) and A549 (lung carcinoma). Senescence was characterized by SA-β-galactosidase (SA-β-gal) activity, proliferation, morphology, and secretion of pro-inflammatory interleukin 6 (IL-6) and interleukin 8 (IL-8). Due to fisetin’s hydrophobic nature, it was encapsulated in liposomes to enhance cellular delivery. Cellular uptake studies confirmed that the liposomes were effectively internalized by both senescent cell types. Treatment with fisetin-loaded liposomes revealed a lack of senolytic effects but showed senomorphic activity, as evidenced by a significant reduction in IL-6 and IL-8 secretion in senescent cells. The liposomal formulation enhanced fisetin’s therapeutic efficacy, showing comparable results even at the lowest tested concentration.

## 1. Introduction

Cellular senescence, first identified by Hayflick et al. in 1961 [1,2], has since then evolved into a significant area of research, particularly concerning its role in cancer. Initially viewed as a protective mechanism due to its ability to induce growth arrest, recent studies over the past years have shown the complex relationship between senescent cells and cancer dynamics [3,4]. Senescent cells can significantly affect cancer progression and metastasis. They may not only halt their own proliferation but also influence neighboring cells through a phenomenon known as the SASP. This secretory profile sends out signals that can alter the tissue microenvironment, potentially fostering conditions conducive to tumor growth. Moreover, there is evidence that senescent cells can re-enter the cell cycle under certain conditions [5,6], which may lead to cancer relapses, with even more aggressive forms of the disease [7,8]. This behavior underscores the dual role of cellular senescence, while it acts as a barrier to tumorigenesis by preventing damaged cells from proliferating, it can also contribute to tumor progression through mechanisms such as SASP. Recent research highlights that therapy-induced senescence (TIS) can involuntarily promote tumorigenesis in surrounding cells. For instance, chemotherapy can lead to an increase in senescent cells in both cancerous and non-cancerous tissues, which may have lasting effects on recovery and the risk of cancer recurrence. The continued presence of these senescent cells can create an immunosuppressive environment, complicating treatment efficacy [9]. While cellular senescence is recognized as a vital tumor-suppressing mechanism due to growth arrest, its potential to promote cancer aggressiveness through various pathways requires a detailed understanding of its role in oncology. This complexity calls for careful consideration in therapeutic strategies aimed at manipulating cellular senescence for cancer treatment.

Senescent cells have anti-apoptotic pathways that protect them from cell death. However, senotherapies, which are therapies specifically targeted to senescence cells, offer promising strategies to eliminate these cells through various mechanisms. Senolytic agents that target and disrupt the anti-apoptotic pathways in senescent cells, induce a process known as senolysis, which leads to the apoptosis of these cells. On the other hand, senomorphic drugs modulate the secretion of factors associated with the SASP, thereby limiting its impact on the surrounding cellular microenvironment. One of the most well-known senotherapeutic agents is fisetin, which is widely recognized in the literature as a senolytic agent, but its activity appears to be cell-type dependent [10,11]. Fisetin works through multiple pathways, including the inhibition of anti-apoptotic proteins like BCL-2 and BCL-xL that promote senolysis, as well as the modulation of inflammatory signaling pathways such as NF-κB and mTOR that contribute to SASP reduction [12,13].

The purpose of a drug delivery system (DDS) is to enhance the efficacy and safety of pharmaceutical substances by controlling the rate, timing, and location of their delivery. Recent advancements in nanotechnology have significantly contributed to the development of innovative drug delivery systems, enabling more targeted and efficient treatment options, minimizing side effects on off-target cells, tissues, or organs [14]. Research on nanosystems has demonstrated that delivering a drug in an encapsulated form can lead to a markedly larger therapeutic effect compared to administering the same concentration of the drug in its free form [15]. Muñoz-Espín’s work utilized a galactose coating on porous silica scaffolds, where high level of β-galactosidase in senescent cells can digest the sugar coating of the beads, enabling drug release only in senescent cells [16]. A similar method utilized by Hou et al. described encapsulation of fisetin in protein isolate with sugar coating. In their work, they noticed that the fisetin alleviates the effects of cellular senescence [17]. Other researchers also used different types of senotherapeutics in liposomal drug delivery systems. For example, Nguyen et al. used rapamycin encapsulated in PEGylated liposomes with conjugated CD9 monoclonal antibody for targeted delivery [18]. In another work, the same group used the same method for targeting senescent cells but used lactose-wrapped calcium carbonate nanoparticles loaded with rapamycin [19].

Liposomes are lipid drug delivery systems that have gained significant attention in the biomedical field due to their unique structural and functional properties. These vesicles are composed of phospholipid bilayers that can encapsulate hydrophilic and/or hydrophobic drugs [20]. The ability of liposomes to encapsulate a wide range of drugs enhances their utility in delivering medications effectively to target sites within the body. Liposomes are known for their general biocompatibility, meaning they can be safely introduced into the body without generating significant immune responses. Despite their potential, the transition of liposomal technologies from laboratory settings to clinical applications has been gradual, primarily due to challenges in production, optimization, and high costs [21,22].

Here we 36focus on using senescent cells, whose senescence was induced with DOX, a commonly used chemotherapeutic, to analyze the effect of fisetin encapsulated in liposomes. During our research we took into consideration the senolytic and senomorphic potential properties of fisetin on senescent cells, investigated their influence, and compared the effect of fisetin in its free form to encapsulated in liposomes. Two lung cell lines were investigated: Human lung adenocarcinoma cells (A549) and healthy lung fibroblasts (WI38). The choice of these two cell lines will allow us to compare senescence induction and fisetin action in a healthy and tumoral line, and provide an initial assessment of the potential therapeutic action of the liposomal formulation of fisetin in cancer treatment.

## 2. Results

### 2.1. Induction and Identification of Cellular Senescence

**Figure 1:**
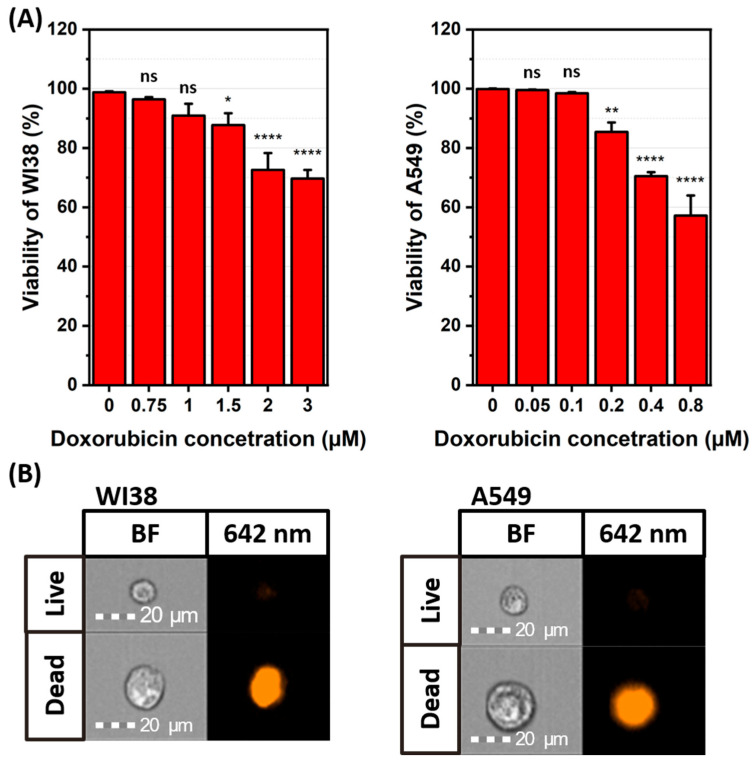
Cytotoxic effect of doxorubicin concentrations used for senescence induction on WI38 and A549. (**A**) the percentage of viable cells with (**B**) representative images of live (no fluorescent) and dead (fluorescent, orange) cells for both cell lines. ns—*p* > 0.05, *–*p* ≤ 0.05, **–*p* ≤ 0.01, ****—*p* ≤ 0.0001.

Human lung adenocarcinoma cells (A549) and healthy lung fibroblasts (WI38) were exposed to varying concentrations of DOX and analyzed for viability, SA-β-gal presence, and proliferation ability to determine the optimal DOX concentration for further experiments. Live/dead assays can distinguish dead cells based on membrane integrity, as the fluorescent signal in this experiment is only visible in cells with compromised membranes. To achieve a large population of senescent cells, DOX concentrations of 0.4 and 0.8 μM for A549 cells and 2 and 3 μM for WI38 were excluded from additional analysis, as these high doses resulted in viability below 80%, as shown on Figure 1. These high doses may induce excessive cell death instead of senescence. Cells maintained under standard conditions were used as controls and identified as non-senescent.

**Figure 2:**
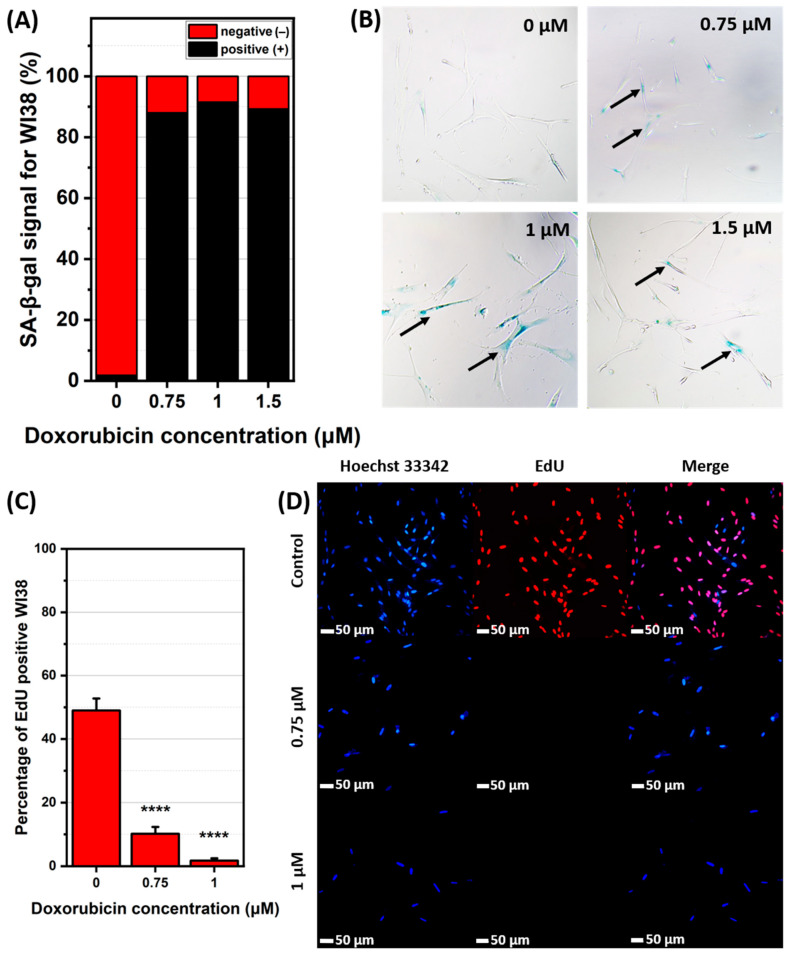
Senescent features of WI38 cell line. (**A**) percentage of SA-β-gal-positive and negative cells with (**B**) representative images of stained cells for control and selected concentrations of doxorubicin used for cellular senescence induction. SA-β-gal-positive cells are pointed out with black arrows (Full size images are in Figure S2). (**C**) percentage of cells actively proliferative and (**D**) representative images of nuclei of all cells in population stained with Hoechst 33342 (blue); proliferative cells were stained with EdU (red) and both images merged together. ****—*p* ≤ 0.0001.

**Figure 3:**
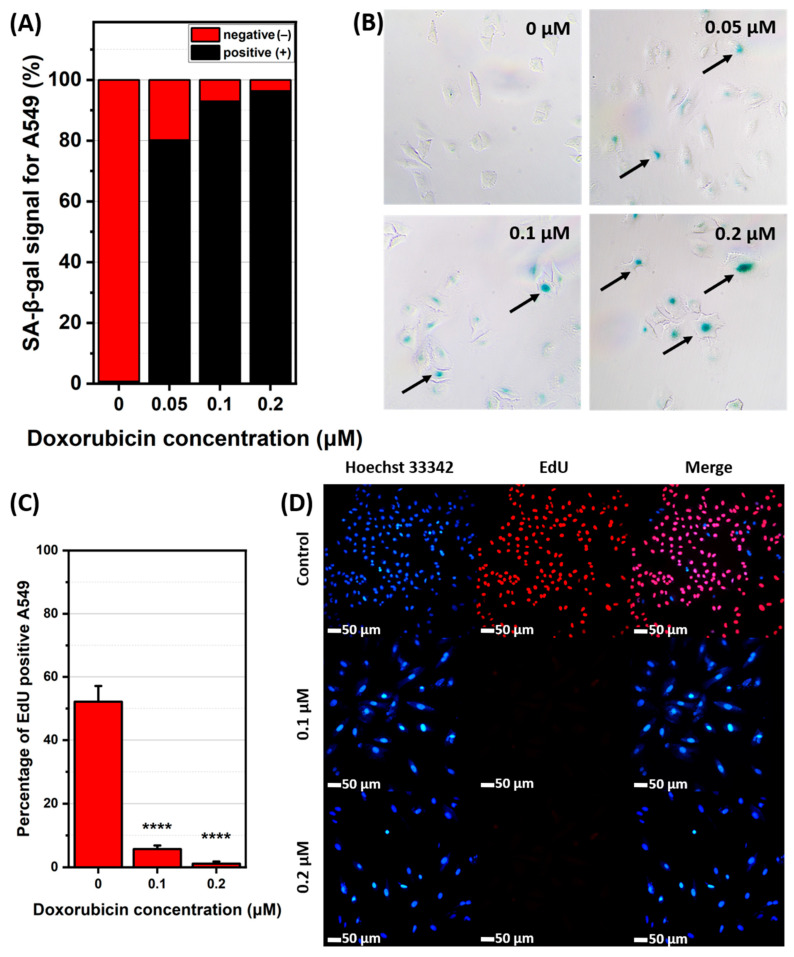
Senescent features of A549 cell line. (**A**) percentage of SA-β-gal-positive and negative cells with (**B**) representative images of stained cells for control and selected concentrations of doxorubicin used for cellular senescence induction. SA-β-gal-positive cells are pointed out with black arrows (Full size images are in Figure S3). (**C**) percentage of cells actively proliferative and (**D**) representative images of nuclei of all cells in population stained with Hoechst 33342 (blue); proliferative cells were stained with EdU (red) and both images merged together. ****—*p* ≤ 0.0001.

The successful senescence induction was validated by SA-β-gal staining, indicating increased β-galactosidase activity in senescent cells. The resulting colorimetric reaction is shown in Figure 2B and Figure 3B. The data indicates that in A549 senescent cells, the percentage of SA-β-gal-positive cells increased with higher DOX concentrations with the most intense staining in A549 cells visible at concentrations of 0.1 and 0.2 μM, shown in Figure 3C. For WI38 cells, the blue staining shows SA-β-gal activity as most prominent at a DOX concentration of 1 μM, while 0.75 and 1.5 μM concentrations exhibited comparable but lower levels, which is visible on Figure 2C. This minor variation is likely due to biological variability within the cell population. Concentrations of 0.1 and 0.2 μM for A549 cells and 0.75 and 1 μM for WI38 cells were selected for further study, as they resulted in the highest percentage of SA-β-gal-positive cells while maintaining a high level of cell viability. Next, the cell proliferation assay was conducted. According to ATCC, the doubling time for both cell lines is approximately 24 h. However, our experimental observations indicated that the doubling time of WI38 cells is closer to 48 h, which is the timeframe we used for this specific cell line. This assay reveals that cells treated with DOX displayed a decrease in division proportional to the increase in drug dosage, as examined by the fluorescence signal of EdU, which only labels actively proliferating cells as presented on Figure 2D and Figure 3D. In A549 cells, EdU-positive signal decreased from 52% in non-senescent cells to 6% (0.1 μM DOX) and 1% (0.2 μM DOX) (Figure 3C). Similarly, in WI38 cells, EdU-positive signal was reduced from 49% to 10% (0.75 μM DOX) and 2% (1 μM DOX) (Figure 2C). Considering the results of viability, SA-beta-gal, and proliferation altogether, the final DOX concentrations were chosen to be 1 μM for WI38 and 0.2 μM for A549.

**Figure 4:**
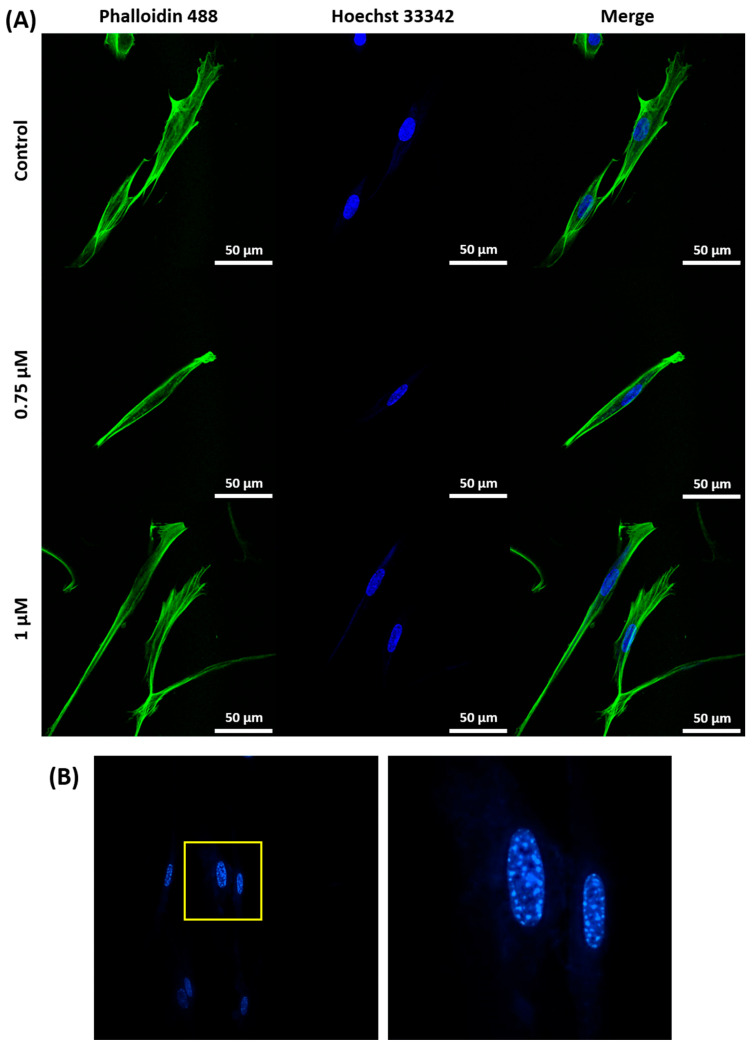
Morphological and nuclear features of senescent WI38 cells. (**A**) Representative fluorescent images showing the morphology of senescent WI38 cells in comparison to untreated control cells. Actin filaments were stained with phalloidin-488 (green), and cell nuclei were counterstained with Hoechst 33342 (blue). The final panel presents a merged view of both channels. Scale bar: 50 μm. (**B**) Hoechst 33342 staining reveals the presence of SAHF, represented by lighter dots on the nuclei structure (the image from yellow box is presented on the right to help visualize SAHF).

**Figure 5:**
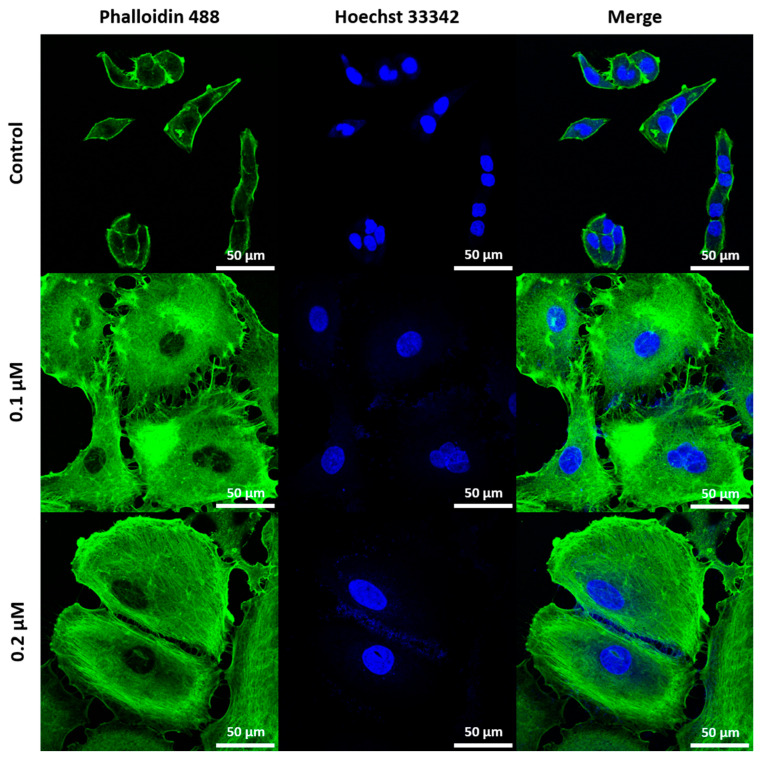
Morphological features of senescent A549 cells. Representative fluorescent images showing the morphology of senescent A549 cells in comparison to untreated control cells. Actin filaments were stained with phalloidin-488 (green), and cell nuclei were counterstained with Hoechst 33342 (blue). The final panel presents a merged view of both channels.

**Figure 6:**
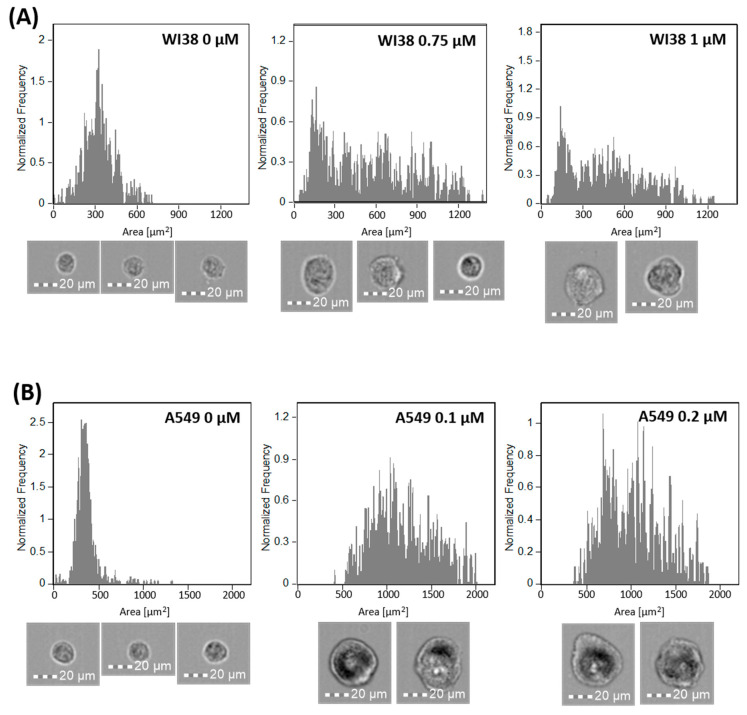
Histograms showing size differences between senescent and untreated (**A**) WI38 and (**B**) A549 cells, measured by imaging flow cytometry. Representative brightfield images of analyzed cells are shown below the corresponding histograms.

Cell morphology was analyzed using confocal laser scanning microscopy (CLSM), revealing distinct changes associated with senescence in both cell lines, as presented on Figure 4 and Figure 5. A549 cells displayed an increase in cell size, shown in Figure 5, confirmed by flow cytometry as shown in Figure 6B, making cell size a reliable marker for senescence in this cell line. While senescent WI38 cells displayed some size increase, it was less consistent, with a subset of senescent cells remaining similar in size to their non-senescent counterparts, which is visible on Figure 6A. However, senescence in WI38 cells was distinguished by the presence of senescence-associated heterochromatin foci (SAHF) within the nucleus, a feature which was not observed in senescent A549 cells, as presented on Figure 4B.

**Figure 7:**
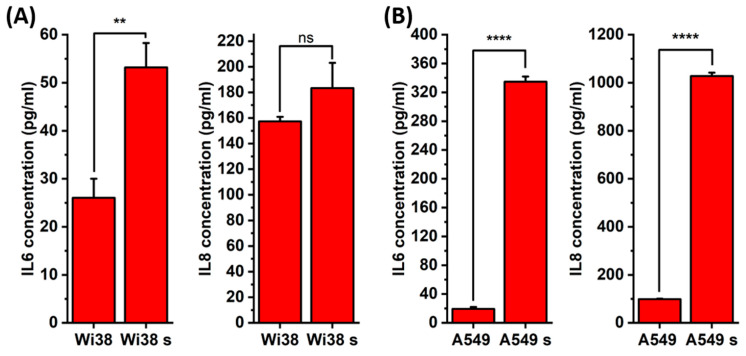
sasp analysis of IL-6 and IL-8 secretion in (**A**) WI38 and (**B**) A549 cells before and after senescence induction. s—senescent, ns—*p* > 0.05, **—*p* ≤ 0.01, and ****—*p* ≤ 0.0001.

To assess the activation of the SASP, we measured the levels of IL-6 and IL-8, in both senescent and non-senescent A549 and WI38 cells using enzyme linked immunosorbent assay (ELISA). IL-6 and IL-8 are interleukins commonly found in SASP across cell lines and induction methods. As both interleukins are proinflammatory and influence the development and progression of cancer, they were chosen for analysis. Senescent A549 cells exhibited a significant increase in both cytokines, with approximately a two-fold higher concentration of IL-6 and a ten-fold higher concentration of IL-8 compared to non-senescent A549 cells, as shown on Figure 7B. Although less pronounced than in A549 cells, senescent WI38 cells also showed elevated levels of IL-6 and IL-8 compared to their non-senescent counterparts, indicating a shift toward a more proinflammatory profile, as visible on Figure 7A.

### 2.2. Characterization of Liposomes

Liposomes were prepared using the thin-layer method, which involves mixing DOPC, DSPE, and cholesterol with an organic solvent, followed by evaporation and rehydration of the lipid residue, and resulting in the formation of liposomes. The proportions of the components were determined based on published protocol by Mignet et al [23]. The liposomes were first analyzed by dynamic light scattering (DLS), as presented in Table 1. Based on the findings, empty liposomes had a hydrodynamic diameter (Z-average) of 115.9 nm ± 0.9 with a polydispersity index (PDI) of 0.155 ± 0.004 and a zeta potential (ζ-potential) of −20.3 mV ± 0.6. Encapsulation of fisetin resulted in a slight reduction in size to 95.1 nm ± 1.0 with a PDI of 0.178 ± 0.008 and a less negative ζ-potential of −11.6 ± 1.2 mV. Stability studies over 30 days showed minimal changes in liposome size for both empty (116.5 nm ± 0.2) and fisetin-loaded liposomes (92.5 nm ± 0.3). While slight increases in PDI (0.181 ± 0.017 and 0.184 ± 0.017, respectively) and decreases in the ζ-potential (−8.25 mV ± 0.54 and −7.0 mV ± 0.3, respectively) were observed, these changes were relatively small, suggesting good stability over time.

**Figure 8:**
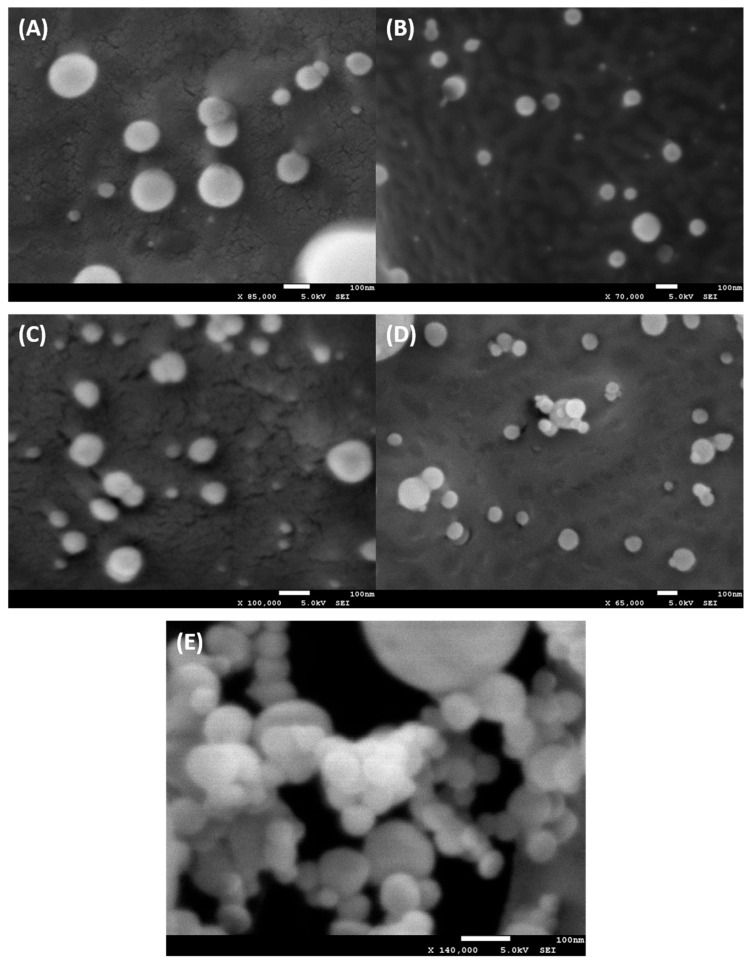
Cryo-SEM images of liposomal formulations. (**A**,**B**) Liposomes without fisetin, (**C**,**D**) liposomes with fisetin encapsulated within their structure, and (**E**) chain-like structures formed by fisetin-encapsulated liposomes. Scale bar = 100 nm. Full size images are in Figures S4–S8.

Cryo-scanning electron microscopy (Cryo-SEM) was used to visualize liposome morphology as presented on Figure 8A–D and compare the size measurements obtained by DLS. The measurements were conducted shortly after liposomes preparation. Both types of liposomes appeared round and regular in shape with smooth surfaces, which corelates to DLS measurements; however, Cryo-SEM revealed that fisetin-loaded liposomes tended to format elongated chain-like structures as visible on Figure 8E.

Fisetin concentration within the encapsulation was determined from fluorescence measurements using a standard lineal calibration curve. After applying the obtained values to a standard linear calibration curve, the fisetin concentration was calculated to be 245 ± 1 μg/mL, corresponding to 850 ± 1 μM. This value was then used to calculate encapsulation efficiency, by using Equation 1, which was found to be 13.68%.

**Figure 9:**
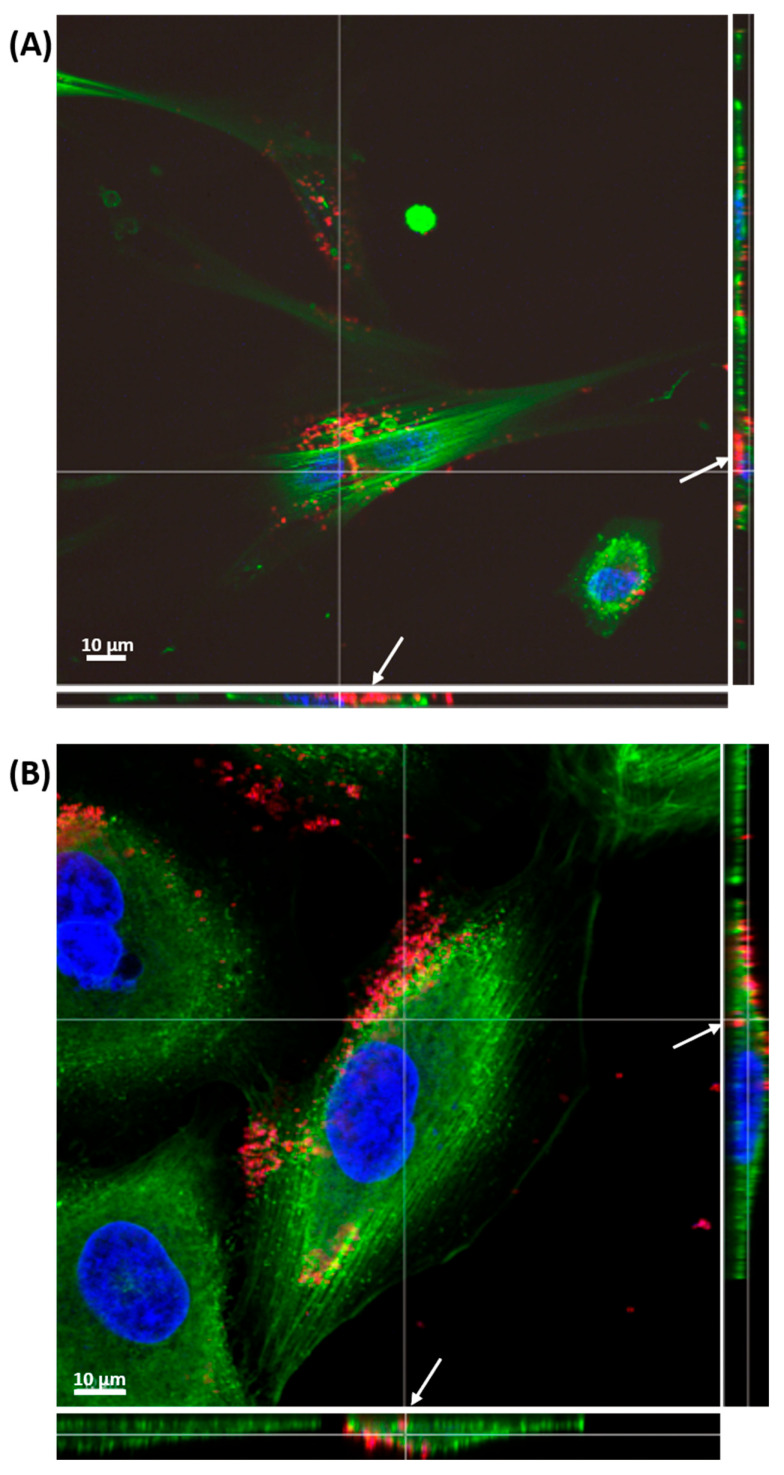
Cellular uptake of Nile red-labeled liposomes. Confocal 3D image showing (**A**) WI38 and (**B**) A549 cells stained with phalloidin-488 (green) to visualize actin filaments and Hoechst 33342 (blue) to label nuclei. Liposomes loaded with Nile red (red). White arrows indicate liposomes internalized by the cell. Scale bar: 10 μm.

Cellular uptake studies using Nile red-labeled liposomes revealed limited internalization into both WI38 and A549 cells after 4 h of incubation. The majority of liposomes were found on the cell surface; however, senescent WI38 cells showed a higher level of liposome internalization (Figure 9A) compared to senescent A549 cells (Figure 9B).

### 2.3. Impact of Fisetin-Encapsulated Liposomes

**Figure 10:**
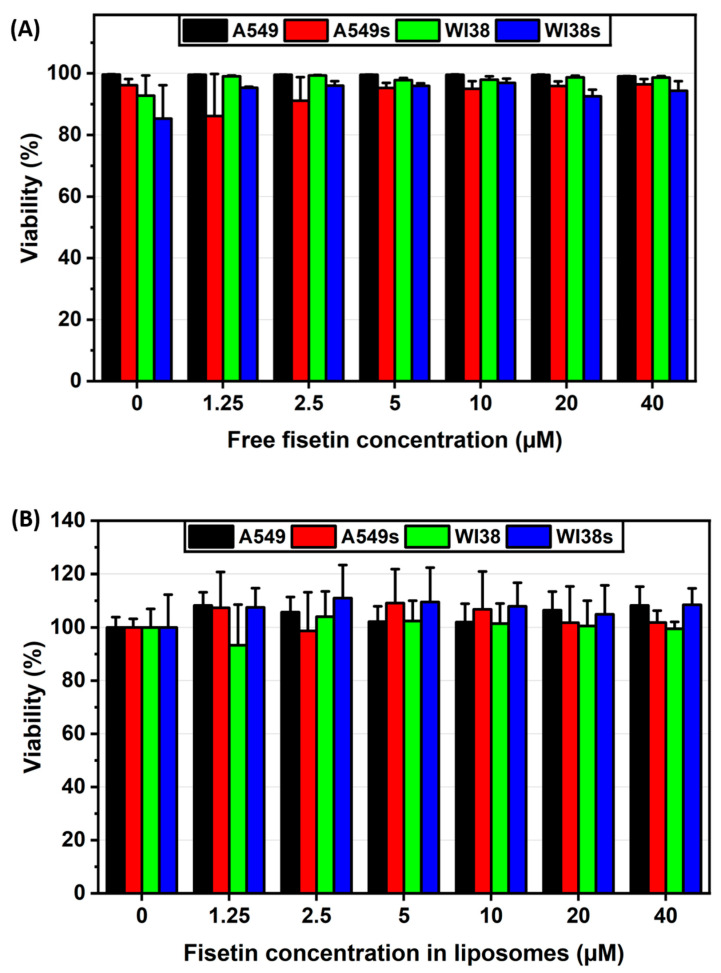
Cell viability following senotherapy. (**A**) Viability of cells after treatment with free fisetin, and (**B**) viability after treatment with fisetin encapsulated in liposomes. s—senescent.

The viability assay revealed that free fisetin did not exhibit specific senolytic properties towards senescent cells as viability decreased at a similar rate in both senescent and non-senescent populations, as presented on Figure 10A. Similarly, fisetin-loaded liposomes also did not induce specific apoptosis in senescent cells, as the effect was similar in both senescent and non-senescent cells, as shown on Figure 10B, indicating a lack of selectivity towards senescent cells.

Live/dead assay images showed no change in cell viability but revealed visible changes in A549 cell shape at higher fisetin concentrations (Figure S9). Based on this, we additionally performed SA-β-gal staining, which showed similar levels of β-galactosidase activity as in control senescent cells (Figure S10). Although cell shape changed, the similar β-galactosidase levels suggest that fisetin may work through other pathways. More studies are needed to understand its full effect.

An additional WST-1 assay was performed to assess the cytotoxicity of free fisetin. The results indicated high cytotoxicity (Figure S11); however, since this assay measures cellular metabolic activity, the findings may reflect fisetin-induced metabolic changes and modulations rather than true cytotoxic effects. This interpretation is supported by the results of previous assays, which did not show comparable cytotoxicity.

**Figure 11:**
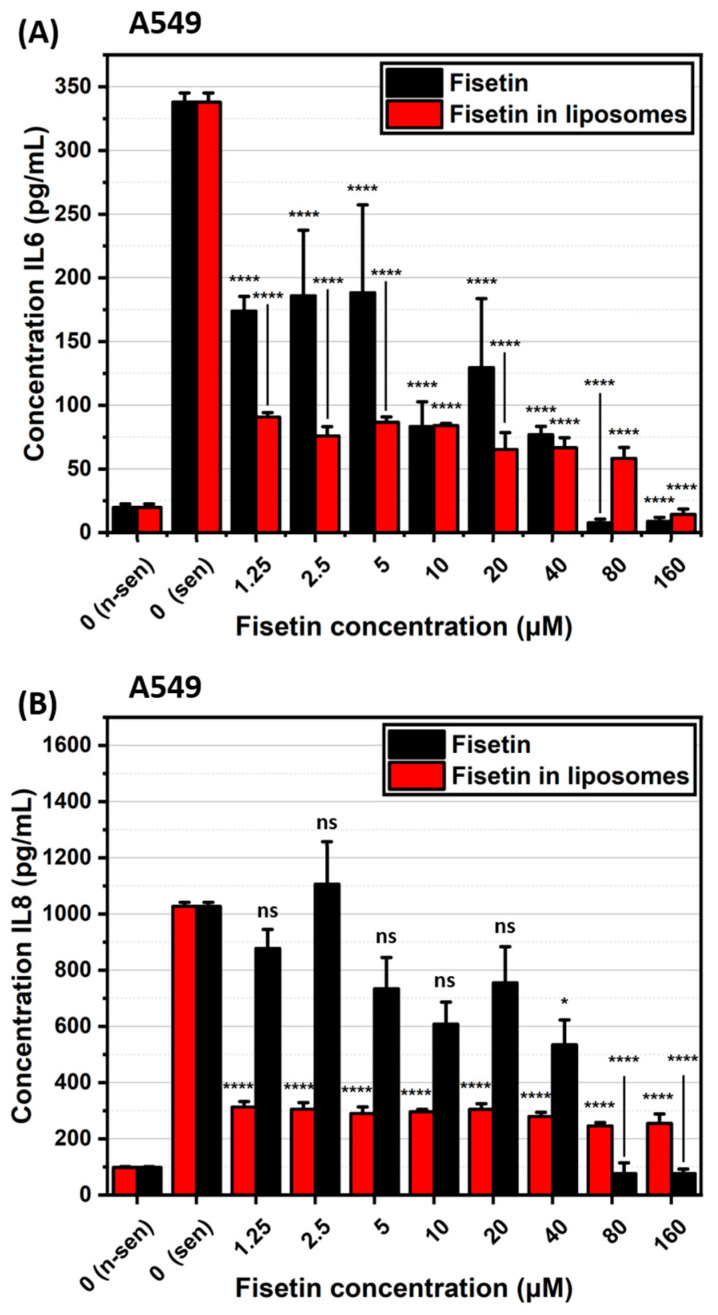
Interleukin concentrations following senotherapy, measured by ELISA. (**A**) IL-6 and (**B**) IL-8 levels in A549 cells. n-sen—non-senescent, sen—senescent. ns—*p* > 0.05, *—*p* ≤ 0.05, and ****—*p* ≤ 0.0001.

**Figure 12:**
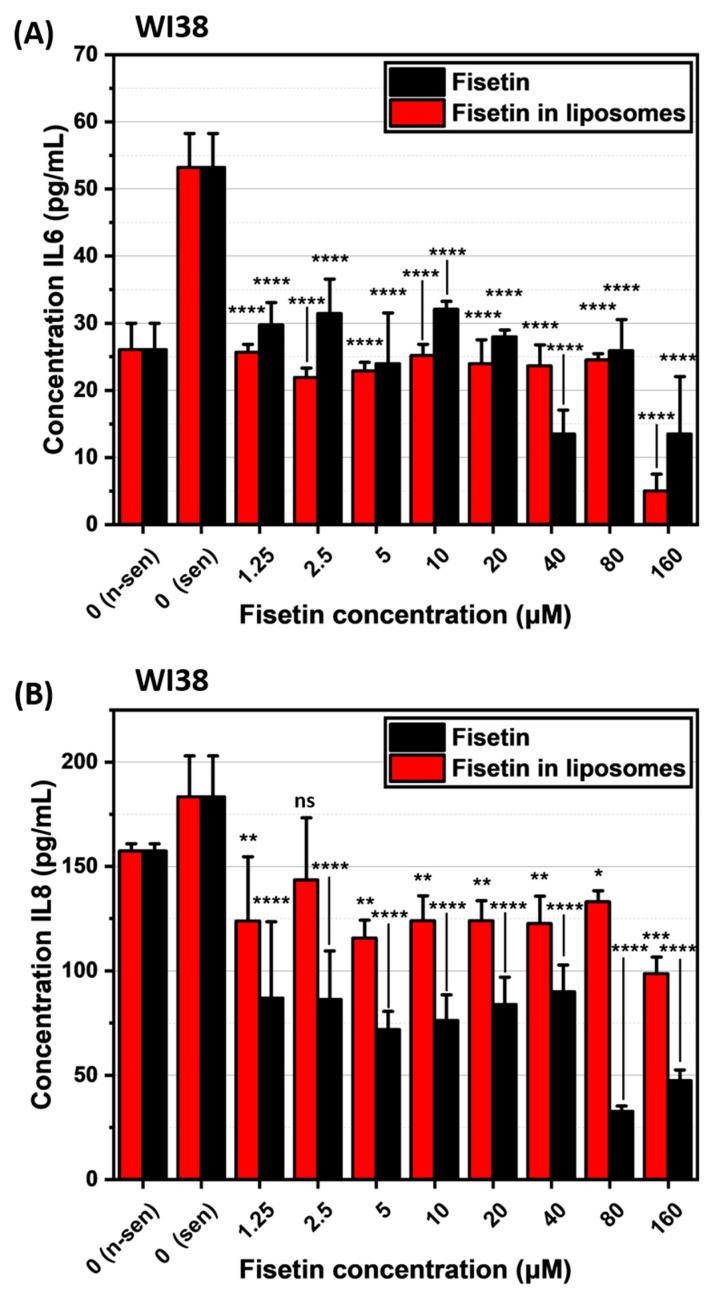
Interleukin concentrations following senotherapy, measured by ELISA. (**A**) IL-6 and (**B**) IL-8 levels in WI38 cells. n-sen—non-senescent, sen—senescent. ns—*p* > 0.05, *—*p* ≤ 0.05, **—*p* ≤ 0.01, ***—*p* ≤ 0.001, and ****—*p* ≤ 0.0001.

ELISA analysis revealed that both free fisetin and fisetin-loaded liposomes have senomorphic properties, meaning they can modulate the SASP in senescent cells. In A549 senescent cells, fisetin-loaded liposomes showed a maximal effect at the lowest concentration tested, reducing the levels of IL-6 (Figure 11A) and IL-8 (Figure 11B), and maintaining this reduction even at higher concentrations. A similar trend was observed in WI38 senescent cells, where fisetin treatment led to a reduction in interleukins levels as presented in Figure 12A,B. In general, the senomorphic effects of fisetin were more pronounced when delivered in liposomes compared to free fisetin.

## 3. Discussion

The main aim of this study was to evaluate the effect of fisetin, a senolytic, both in free form and encapsulated in liposomes, on senescent A549 and WI38 cell lines. Additionally, the study aimed to assess the efficiency of delivering the drug in liposome formulation compared to its free form.

DOX is a well known anthracycline chemotherapeutic agent. It causes DNA intercalation and inhibition of topoisomerase II, leading to the disrupting of DNA replication and transcription, which ultimately leads to the apoptosis of cancer cells. However, its clinical use is limited by multiple side effects such as cardiotoxicity, hepatotoxicity, induction of stem cell-like features in cancer cells, and creating drug resistance during long-time exposures [24,25]. Moreover, it affects healthy cells due to its non-specific nature. Because of its influence on all types of cells, cellular senescence was induced on adenocarcinoma cell line A549 and healthy fibroblasts WI38. This allowed us to compare both cancerous and non-cancerous lung cell responses. Since lung cancer is one of the most common cancers worldwide, according to the World Health Organization (WHO), studying both cell types helps us better understand how fisetin might work as a supportive treatment after chemotherapy. The method of the cellular senescence induction was selected based on the available literature but was found insufficient; therefore, the concentration of DOX was established experimentally. For the induction process, we were using already established protocols. However, in the case of the WI38 cell line, used concentrations of DOX were very wide, from 0.1 μM [26] to 2 μM [27] and none of the suggested concentration was prominent in senescence induction. This required us to establish the concentration experimentally. DOX can induce cellular senescence in both cancerous and non-cancerous cell lines [28]. Senescent cells exhibit shared characteristics, such as SA-beta-gal overexpression, but can also differ in overall morphological and epigenetic changes [29,30].

Here we noticed differences in the overall size of the A549 cells, which were not pronounced in the WI38 cells without the imaging flow cytometry analysis. Bojko et al. conducted excessive investigation on overall morphology of the cells with senescence induced by various methods. Their findings show that each induction method can influence morphology differently on various cell lines [29]. Some researchers started developing systems based on machine learning for senescence detection based on their morphology, but each is prepared for specific cell types and/or cell lines [31,32,33].

However, WI38 cells exhibited another hallmark for senescent cells known as SAHF, which was not detected in A549. Similar results were established by Zhao et al. where they obtained SAHF in senescent WI38 [34]. Kosar et al. investigated the SAHF phenomenon on multiple cell lines using different stress stimuli, where they noticed that SAHF creation is dependable on the induction method and cell line [30]. Although these findings are interesting, further investigation of SAHF was beyond the scope of our study.

Our findings and studies from other researchers emphasize the importance of determining the optimal concentration of stress inducers for each cell line, as the effects can vary based on cell type, tissue origin, and even lab settings and reagents. These factors can also influence the biomarkers presented, which is why using multiple methods to analyze senescence biomarkers is crucial [29,30,35,36].

The preparation of cellular senescence protocol was important for further experiments. Proinflammatory cytokines and chemokines, components of the SASP, are thought to activate epithelial–mesenchymal transition (EMT) and cancer invasiveness, promoting cancer relapses [37]. Senotherapy, which aims to reduce senescent cells or the influence of SASP by reducing its secretion, could be beneficial for cancer patients by reducing the risk of recurrence. Taking into consideration the heterogenous nature of senescent cells, it is very unlikely that the same senotherapeutic will act in the same dosage and in the same manner [10,11]. Fisetin, a flavonoid widely recognized for its senolytic properties, can induce selective apoptosis in senescent cells, reducing their population. However, our research shows that fisetin decreased the secretion of both interleukins in senescent A549 and WI38 without causing selective apoptosis.

Senomorphic action of fisetin has not been studied before on senescent A549 and WI38 cell lines. In general, it was found that fisetin can act on BCL-2 family protein as well as PI3K/Akt pathway, which in both cases leads to apoptosis, but also on mTOR signaling pathway, which is a SASP regulator, which suggests its senomorphic properties [38].

Due to hydrophobicity of fisetin, liposomes allow for the enhanced delivery of fisetin into the cells. We followed an established protocol by Mignet et al. for preparing fisetin-encapsulated liposomes [23], but our analysis of physicochemical properties revealed discrepancies with their results. Notably, the encapsulation efficiency was significantly lower than theirs. This may result from methodological differences. We used a lipid composition without PLGA and removed chloroform using argon instead of rotary evaporation, both of which can influence drug loading.

The stability studies conducted over 30 days showed minimal changes in liposome size for both empty and fisetin-loaded liposomes, with slight increases in PDI and decreases in zeta potential. These changes are relatively small, indicating good stability over time. However, the Cryo-SEM images revealed that fisetin-loaded liposomes tend to form elongated, chain-like structures, which could influence their interaction with cells. We can assume that the chain-like structures form due to changes in the surface charge of fisetin-loaded liposomes. Their zeta potential dropped by about 8.7 mV compared to unloaded liposomes. Additionally, the negative charge of liposomes could adversely impact liposomes uptake as cell surface is also negatively charged [39,40]. Uptake studies using Nile red-labeled liposomes show limited internalization into senescent WI38 and A549 cells after 4 h of incubation, with most liposomes remaining on the cell surface. Studies show that different cell types have a different uptake of liposomes due to different interaction mechanisms [41,42], which were not studied in this work. The limited overall internalization may be attributed, as mentioned, to liposome size, composition, or surface characteristics.

We examined the impact of fisetin on senescent A549 and WI38 cells, both in its free form and encapsulated in liposomes. Although fisetin did not exhibit senescence-specific cytotoxicity [10,11], we observed a significant reduction in IL-6 and IL-8 levels for both cell lines compared to senescent cells that were not treated with fisetin. In case of fisetin encapsulated in liposomes, the concentration of IL-6 and IL-8 were at similar, low levels across all tested concentrations, even at the lowest dose when compared to untreated senescent cells. This presents the power of liposomes as a drug delivery system, as it improved the efficiency of the drug in our in vitro studies.

To conclude, this study successfully prepared cellular senescence induction protocol and validated its effectiveness via analysis of various senescence biomarkers. It was also shown how heterogenous senescent cells are between cell lines. The treatment of senescent cells with fisetin showed its senomorphic properties without causing selective apoptosis. Liposomes formulated with encapsulated fisetin were successfully used as drug delivery systems, with the efficient senomorphic effects of the drug in reducting interleukin secretion compared to its free form. Further studies will involve the combination of fisetin with other senolytics achieve selective apoptosis in cancer cells together with senomorphic effects.

## 4. Materials and Methods

### 4.1. Materials

All chemicals and plastics were purchased from Merck Life Sciences (Darmstadt, Germany) unless otherwise specified. Fetal bovine serum (FBS) was obtained from biowest (Nuaillé, France). Doxorubicin was purchased from LC Laboratories (Woburn, MA, USA). Hank’s Balanced Salt Solution, LIVE/DEAD™ Fixable Far Red Dead Cell Stain Kit, Hoechst 33342, 16% Formaldehyde Methanol-free, EthD-2, calcein AM were purchased from Thermo Fisher Scientific (Waltham, MA, USA). ClickTech EdU Cell Proliferation Kit for Imaging was purchased from baseclick GmbH (Neuried, Germany). Phalloidin-iFluor 488 Reagent was purchased from abcam (Cambridge, UK). Human IL-6 DuoSet ELISA, Human IL-8/CXCL8 DuoSet ELISA, and DuoSet ELISA Ancillary Reagent Kit were purchased from R&D Systems (Minneapolis, MN, USA). 1,2-dioleoyl-sn-glycero-3-phosphocholine (DOPC) and 1,2-distearoyl-sn-glycero-3-phosphoethanolamine (DSPE) were purchased from Avanti Polar Lipids (Alabaster, AL, USA).

### 4.2. Cell Cultures

A549 and WI38 cell lines were purchased from ATCC (Manassas, VA, USA) and maintained in EMEM enriched with 10% FBS, 1% sodium pyruvate, and 1% antibiotics (penicillin 100 μg/mL, streptomycin 100 μg/mL) under standard conditions (37 °C, 5% CO_2_).

### 4.3. Senescence Induction

A549 and WI38 cell lines were cultured in T25 flasks (Greiner Bio-One, Kremsmünster, Austria) at a density of 1.5 × 10^5^ cells and incubated for 24 h to allow attachment. Senescence was induced using DOX: A549 cells were treated with a two-fold serial dilution of DOX ranging from 0.05 to 0.8 μM, while WI38 cells were exposed to two separate dilution ranges: 0.75 to 3 μM and 1 to 2 μM. Each treatment lasted for 72 h, after which cells were washed with Hank’s Balanced Salt Solution (HBSS) to remove residual DOX. Fresh drug-free medium was added, and cells were incubated for an additional 72 h to allow recovery before further analysis.

### 4.4. Live/Dead Cell Viability Assay

Cell viability after DOX treatment was assessed using the LIVE/DEAD™ Fixable Far Red Dead Cell Stain Kit based on membrane integrity. Senescent and control cells were trypsinized and collected into 1.5 mL centrifuge tubes, washed with phosphate-buffered saline (PBS), and stained with 1 μL of far-red reactive dye in 1 mL Dulbecco’s phosphate-buffered saline (DPBS) for 30 min at RT in the dark. Cells were then fixed with 4% formaldehyde in PBS for 15 min at RT, washed, and resuspended in 50 μL PBS. Samples were analyzedon FlowSight imaging flow cytometer (Amnis Luminex, Austin, TX, USA), using a 642 nm laser at low flow rate, acquiring 1000 events per sample in triplicate. Data and images were processed using IDEAS^®^ 6.3 software.

### 4.5. SA-β-Galactosidase Staining

Senescent and control cells were seeded onto Nunc™ Lab-Tek™ chamber slides (Roskilde, Denmark) at densities of 4.7 × 10^4^ for A549 and 5.6 × 10^4^ for WI38 cells per well and incubated for 24 h. Senescence Cell Histochemical Staining Kit was used for SA-β-gal staining, in accordance with the manufacturer’s instructions. SA-β-gal staining incubation times for both cell lines were determined experimentally, approximately 5 h and 15 h for WI38 and A549, respectively. Following staining, cells were washed with PBS and counterstained with 16 μM Hoechst 33342 for 10 min at RT in the dark to visualize the nuclei. Imaging was performed using a FV1000 CLSM (Olympus, Tokyo, Japan) in both bright-field (SA-β-gal) and fluorescence modes with 405 nm excitation (425–475 nm emission) (nuclei). ImageJ 1.54j was used to quantify total and SA-β-gal-positive cells for statistical analysis.

### 4.6. EdU Cell Proliferation Assay

Cell proliferation was assessed using the ClickTech EdU assay following the manufacturer’s protocol. After the induction procedure, A549 and WI38 cells were seeded in 96-well black plates (Greiner Bio-One, Kremsmünster, Austria) at 2 × 10^3^ cells/well density. Cells were incubated with 200 μL of EdU working solution at 20 μM concentration for doubling time, which was measured experimentally (A549: ~24 h, WI38: ~48 h). Cells were fixed with 4% formaldehyde in PBS for 15 min at RT, permeabilized with 0.1% Triton X-100 for 20 min at RT, and labeled with staining solution (Eterneon-Red 645 Azide) for 30 min at RT. Hoechst 33342 was used for nuclear staining. Images were captured using the IN Cell Analyzer 2000 (GE Healthcare Life Sciences, Marlborough, MA, USA.) with DAPI (blue) and Cy5 (red) filters (20 fields/well at 20x magnification) and analyzed with IN Cell Developer Toolbox 1.9.3 and OriginPro 2024.

### 4.7. Morphology Evaluation

Senescent and non-senescent A549 and WI38 cells were seeded on Nunc™ Lab-Tek™ 8-well chamber slides (1 × 10^4^ cells/well) and incubated overnight. Cells were fixed with 4% formaldehyde in PBS, permeabilized with 0.1% Triton X-100, blocked with 5% bovine serum albumin (BSA), and stained with Phalloidin-iFluor 488 for actin filaments and Hoechst 33342 for nuclei. Imaging was conducted using an FV1000 CLSM (Olympus, Tokyo, Japan) with a 60× objective (1.4 oil immersion lens). Actin was visualized using 488 nm excitation (495–545 nm emission); nuclei with 405 nm excitation (425–475 nm emission).

To analyze the size of the cells, senescent and non-senescent A549 and WI38 cells were seeded in T25 flasks at 1.5 × 10^5^ density and treated as previously described in triplicates. After trypsinization and centrifugation (1200 rpm, 5 min), cells were resuspended in 100 μL PBS and analyzed using a FlowSight flow cytometer (Amnis Luminex, Austin, TX, USA) in bright field mode at the lowest flow rate (1000 events/sample). Cell size was assessed with IDEAS^®^ software using consistent gating based on area and aspect ratio. Histograms were created to display cell area in μm^2^.

### 4.8. Interleukin Level Evaluation

To quantify IL-6 and IL-8 levels, ELISA was performed following the manufacturer’s instructions. Senescent and non-senescent A549 and WI38 cells were seeded in 96-well plates (2 × 10^3^ cells/well). After 24 h in fresh medium, supernatants were collected for analysis. Plates were coated overnight with capture antibodies, blocked for 1 h, then incubated with standards, blanks, and samples for 2 h. Detection antibodies and streptavidin-HRP were sequentially added, with 2 h and 20 min incubations, respectively. Plates were washed between steps, and the reaction was stopped with a stop solution. Absorbance was measured at 470 nm with 570 nm as reference using an Anthos Zenyth 340rt microplate reader (Biochrom Ltd, Cambridge, UK).

### 4.9. Liposomes Formulation

Liposomes were prepared using the thin-film hydration method adapted from Mignet et al., with modifications. DOPC (20.7 mg/mL), DSPE (10 mg/mL), and cholesterol (10 mg/mL) were dissolved in chloroform [23]. For fisetin-loaded liposomes, fisetin was dissolved in absolute ethanol (1.7 mg/mL) and added to the lipid mixture. Lipid ratios used for formulation are presented in Table 2. The solvent was evaporated under an argon stream, leaving a thin lipid film on the glass walls.

The lipid film was rehydrated with HEPES buffer (8 mg/mL final lipid concentration) and incubated for 24 h at 4 °C. The suspension was extruded through polycarbonate membranes (Ø 800, 400, 200, 100 nm) to obtain final uniform liposomes at a size close to 100 nm. Fisetin-loaded liposomes were purified via Sephadex G-25 column (Cytiva, Marlborough, USA) pre-equilibrated with 25 mL HEPES. A total of 2.5 mL liposome–fisetin suspension was filtered and eluted with 3.5 mL HEPES, yielding 6 mL of purified liposomes at 3.33 mg/mL.

Fisetin concentration in liposomes was determined using a fluoroSENS spectrofluorometer (Gilden Photonics, Clydebank, UK). Maximum excitation/emission wavelengths were detected at 418/486 nm. Liposomes were dissolved in methanol at 1:50 ratio, and empty liposomes were used for background subtraction. A standard curve was generated using fisetin in methanol (50–1000 ng/mL). Fluorescence readings were applied to the calibration curve to calculate encapsulated fisetin concentration. Encapsulation efficiency (EE%) was calculated according to the formula:

$$EE\%\;=\;\frac{M_{L}}{M_{T}}\;\times \;100\%$$

where $M_{L}$ is mass of drug in liposomes and $M_{T}$ is total mass of drug used in formulation.

### 4.10. Liposome Characterization

DLS was employed using a Zetasizer Nano (Malvern, Worcestershire, UK) to determine Z-average, PDI, and ζ-potential of the liposomes with and without fisetin. Samples, 10 μL of liposomes in 990 μL of distilled water, were analyzed in folded capillary cells, and results are presented as mean ± SD from three measurements.

Cryo-SEM was used to examine liposome morphology using JEOL 7001F SEM with a PP3000T Cryo System (Quorum Technologies, Laughton, UK), following the PP3000T User Manual v1.4. About 30 μL liposome suspension was plunge-frozen in nitrogen slush (−210 °C), fractured, sublimated at −90 °C for 35 min, and sputter-coated with platinum (10 mA, 100 s). Images were captured at 5 keV under cryogenic conditions (−190 ± 2 °C, ~2.6 × 10^−5^ Pa).

### 4.11. Cellular Uptake of Liposomes

For cellular uptake analysis, liposomes were modified during their formulation with addition of Nile red dye in ethanol (1 mg/mL) by adding 1 μL per 500 μL of final solution of liposomes. Next, liposomes were added to cells, which were priorly seeded on 8-well Lab-Tek chamber slides at density 1 × 10^4^ cells/well, and incubated with Nile red-labeled liposomes (300 μg/mL) for 4 h. After incubation, cells were stained with Phalloidin-iFluor 488 and Hoechst 33342 as previously described. Imaging was performed using the FV1000 CLSM (Olympus, Tokyo, Japan), with Nile red detected using 638 nm excitation and 650–670 nm emission filters.

### 4.12. Cell Viability Assay After Fisetin Treatment

To evaluate the senolytic effect of fisetin-loaded liposomes, A549 and WI38 senescent and non-senescent cells were seeded in black 96-well plates at 2 × 10^3^ cells/well. Cells were treated with fisetin in free and liposomal form, both at concentration ranges between 12.5 and 160 μM in triplicates. Fisetin concentration in liposomes was determined via fluorescence and matched to a calibration curve (see Supplementary Figure S1 for liposome–fisetin correlation). After 48 h, cells were stained with 2 µM calcein AM, 2 µM EthD-2, and 8 μM Hoechst 33342 in DPBS (100 µL/well) for 30 min at 37 °C. Imaging was performed using the IN Cell Analyzer 2000 (GE Healthcare Life Sciences, Marlborough, MA, USA) with Cy5 (red, EthD-2), DAPI (blue, Hoechst 33342), and FITC (green, calcein AM) filters (10 fields/well at 20× magnification). Cell counts and viability percentages were analyzed using IN Cell Developer Toolbox and MS Excel.

### 4.13. Interleukin Evaluation After Fisetin Treatment

To evaluate the senomorphic effect of fisetin, senescent A549 and WI38 cells were seeded in 96-well plated at density 2 × 10^3^ cells/well and treated with increasing concentration of fisetin-loaded liposomes as described previously. After 48 h, cells were washed to remove residual liposomes and incubated for an additional 24 h in fresh, drug-free medium. Next, collected medium was then analyzed by ELISA to determine IL-6 and IL-8 levels, based on freshly prepared calibration curve and measurement parameters described earlier.

### 4.14. Statistical Analysis

All experiments were conducted in triplicate, and results are presented as mean ± standard deviation. ELISA results and all graphs were analyzed and prepared using OriginPro 2024.

## Figures and Tables

**Table 1:** Results of DLS measurement for empty liposomes and fisetin-encapsulated liposomes at two time points, at day 1 and day 30 after storage in 4 °C.

	Day 1			Day 30		
	Z-Average nm	PDI	ζ-Potential mV	Z-Average nm	PDI	ζ-Potential mV
Liposomes	115.9 ± 0.9	0.155 ± 0.004	−20.3 ± 0.6	116.5 ± 1.0	0.181 ± 0.017	−8.3 ± 0.5
Liposomes + Fisetin	95.1 ± 0.9	0.178 ± 0.008	−11.6 ± 1.2	92.5 ± 0.3	0.184 ± 0.017	−7.0 ± 0.3

**Table 2:** Proportions of the components used in the formulations of liposomes (wt%).

	Fisetin Encapsulated Liposomes	Empty Liposomes
DOPC	78	80.4
DSPE	13	13.4
Cholesterol	6	6.2
Fisetin	3	-

## Data Availability

The datasets generated and/or analyzed during the study are available from the corresponding authors upon reasonable request.

## Supplementary Materials

The following supporting information can be downloaded at: https://www.mdpi.com/article/10.3390/ijms26157489/s1.
