# Supplementary material for: Targeting Cellular Senescence with Liposome-Encapsulated Fisetin: Evidence of Senomorphic Effect
Source: Int J Mol Sci. 2025 Aug 2;26(15):7489. doi: 10.3390/ijms26157489 (PMC12347707; doi:10.3390/ijms26157489)
Supplement: Supplementary file 1 [file ijms-26-07489-s001.zip › ijms-3741006-supplementary.pdf]

Supplementary materials

Targeting Cellular Senescence With Liposome Encapsulated Fisetin: Evidence of Senomorphic Effect

Agata Henschke[1]*, Bartosz Grześkowiak[1], Olena Ivashchenko[1], María Celina Sánchez-Cerviño[2], Emerson Coy[1]*and Sergio Moya[3]*.

[1]NanoBioMedical Centre, Adam Mickiewicz University, Wszechnicy Piastowskiej 3, 61-614 Poznan, Poland

[2]Research Institute of Materials Science and Technology, INTEMA (UNMdP-CONICET), Av. Colón 10850, Mar8del Plata B7606BWV, Argentina

[3]Soft Matter Nanotechnology Group, CIC biomaGUNE, Basque Research and Technology Alliance (BRTA), 10Paseo Miramón 182, San Sebastián, Guipúzcoa, 20014 Spain

Corresponding Authors: agata.henschke@amu.edu.pl, coyeme@amu.edu.pl, smoya@cicbiomagune.es

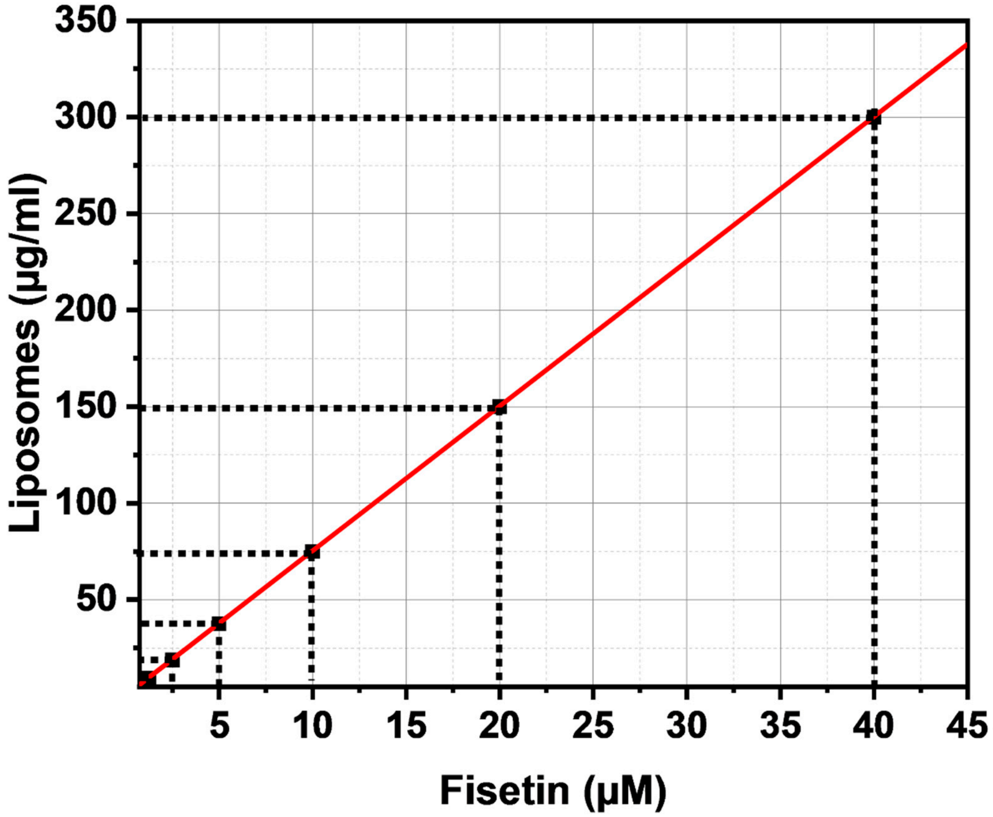

Figure S1. Correlation curve shows how fisetin concentration changes with different liposome concentrations.

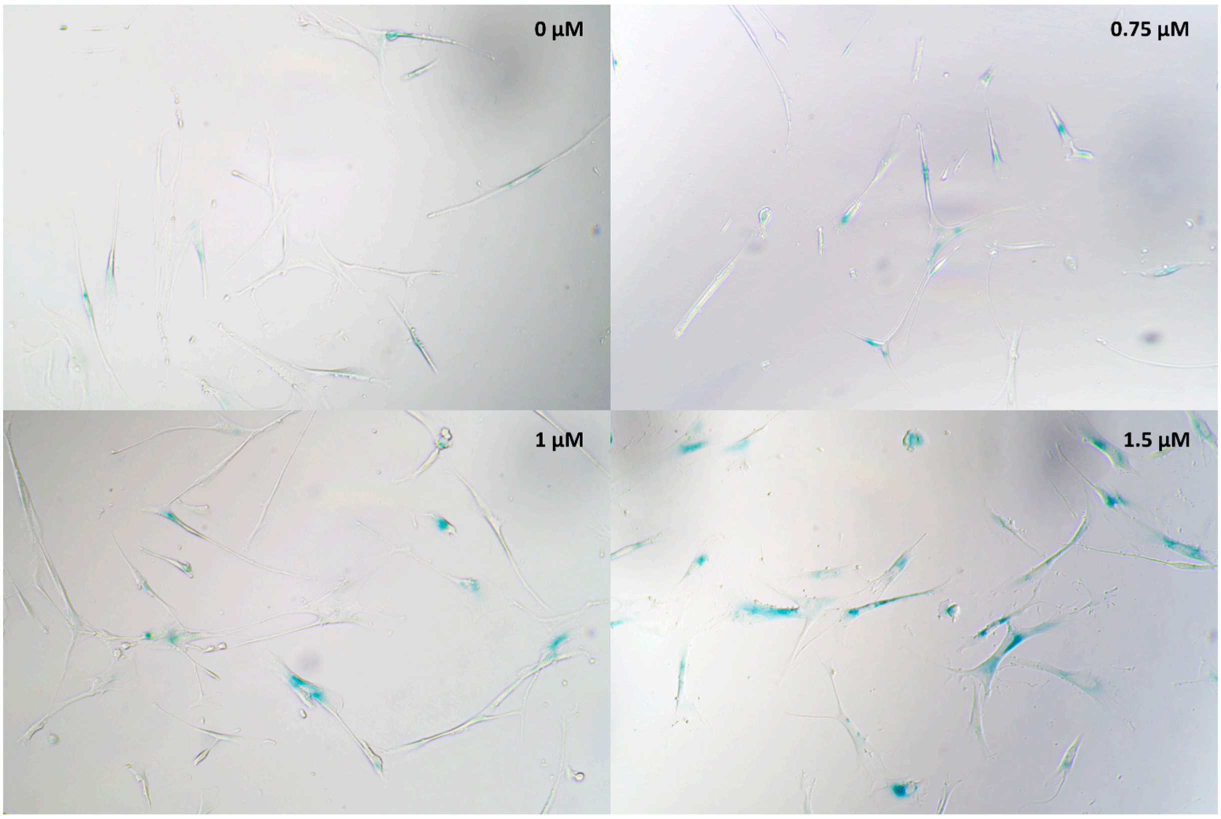

Figure S2. Uncropped version of representative images of stained WI38 cells with SA-β-gal for control and selected concentrations of doxorubicin used for cellular senescence induction

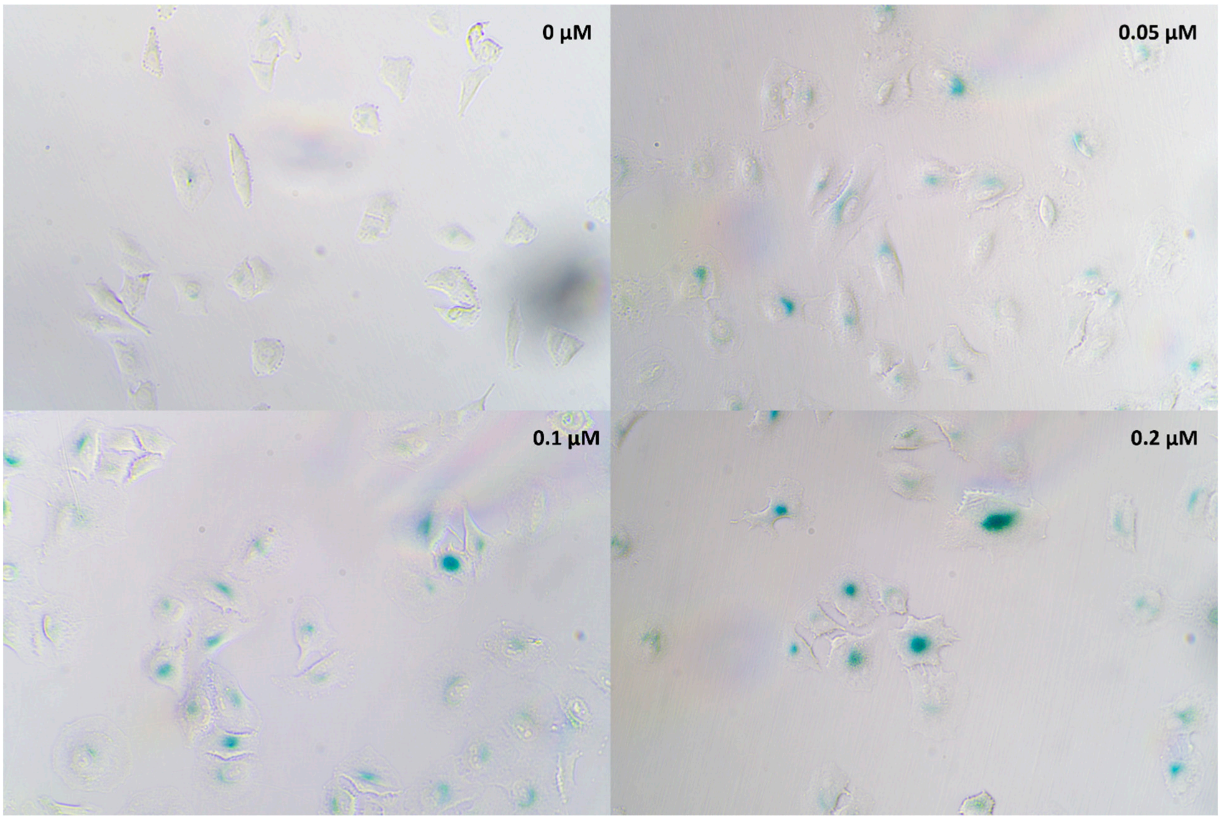

Figure S3. Uncropped version of representative images of stained A549 cells with SA-β-gal for control and selected concentrations of doxorubicin used for cellular senescence induction.

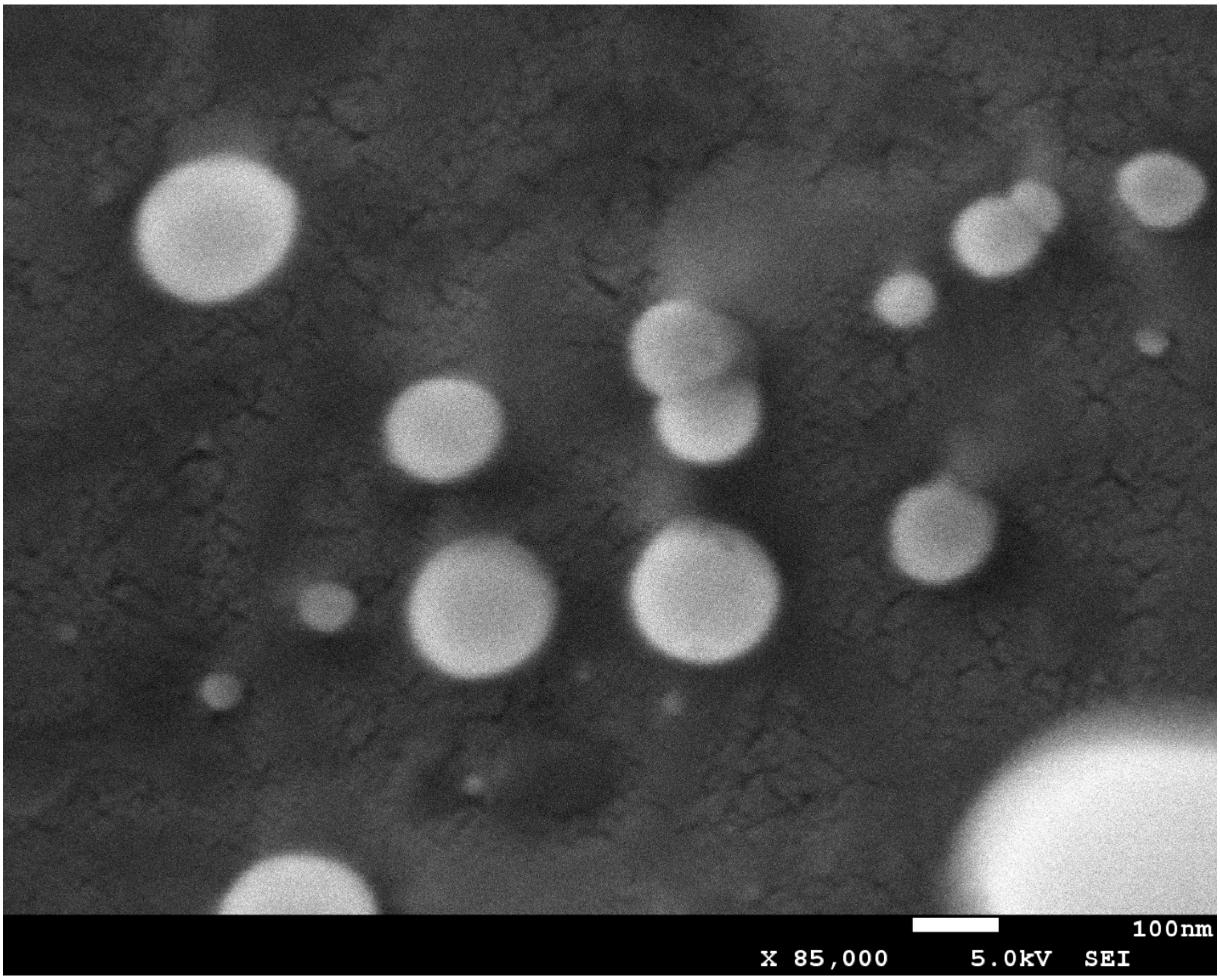

Figure S4. Full size of Cryo-SEM images of liposomal formulations, representation of Figure 8A.

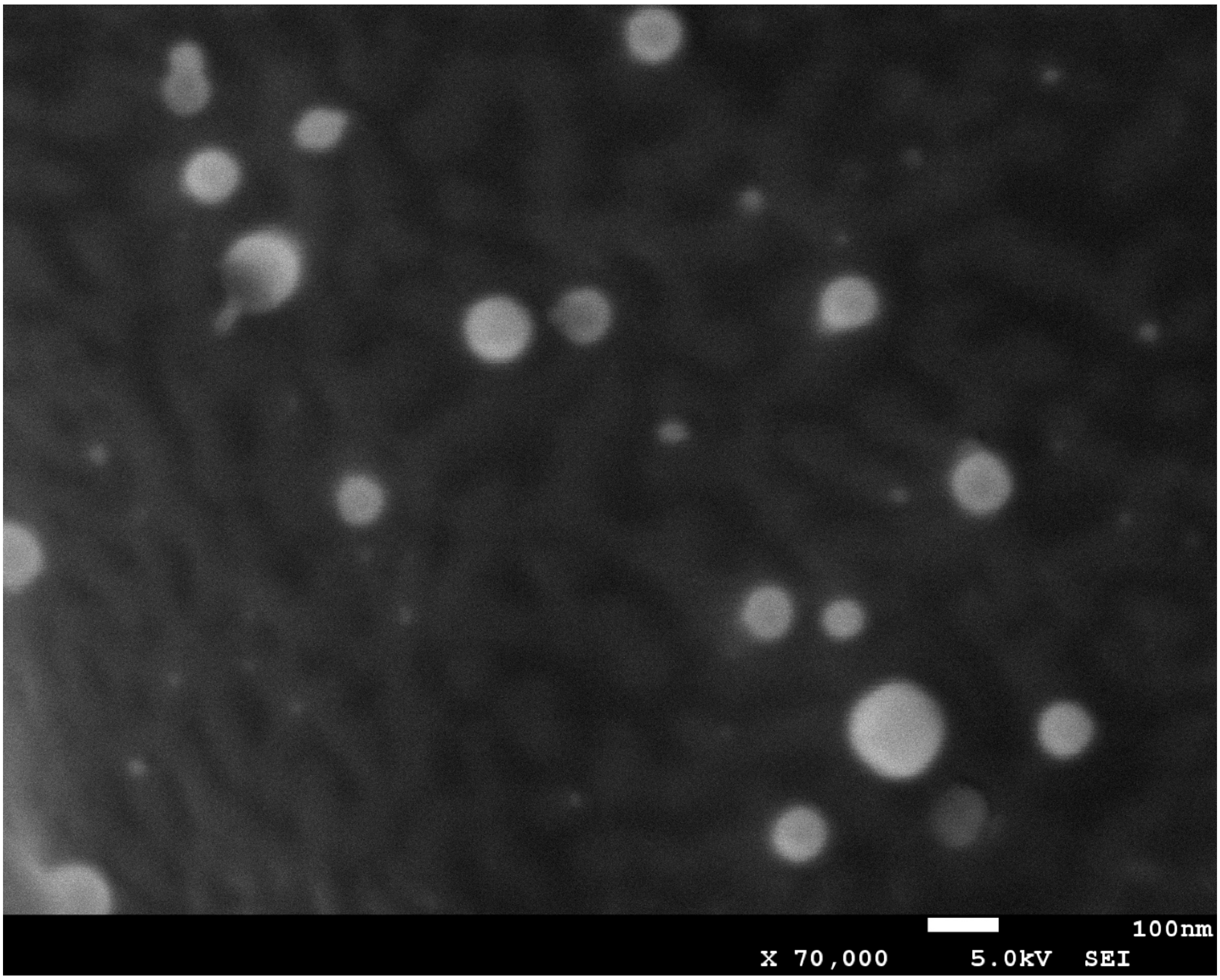

Figure S5. Full size of Cryo-SEM images of liposomal formulations, representation of Figure 8B.

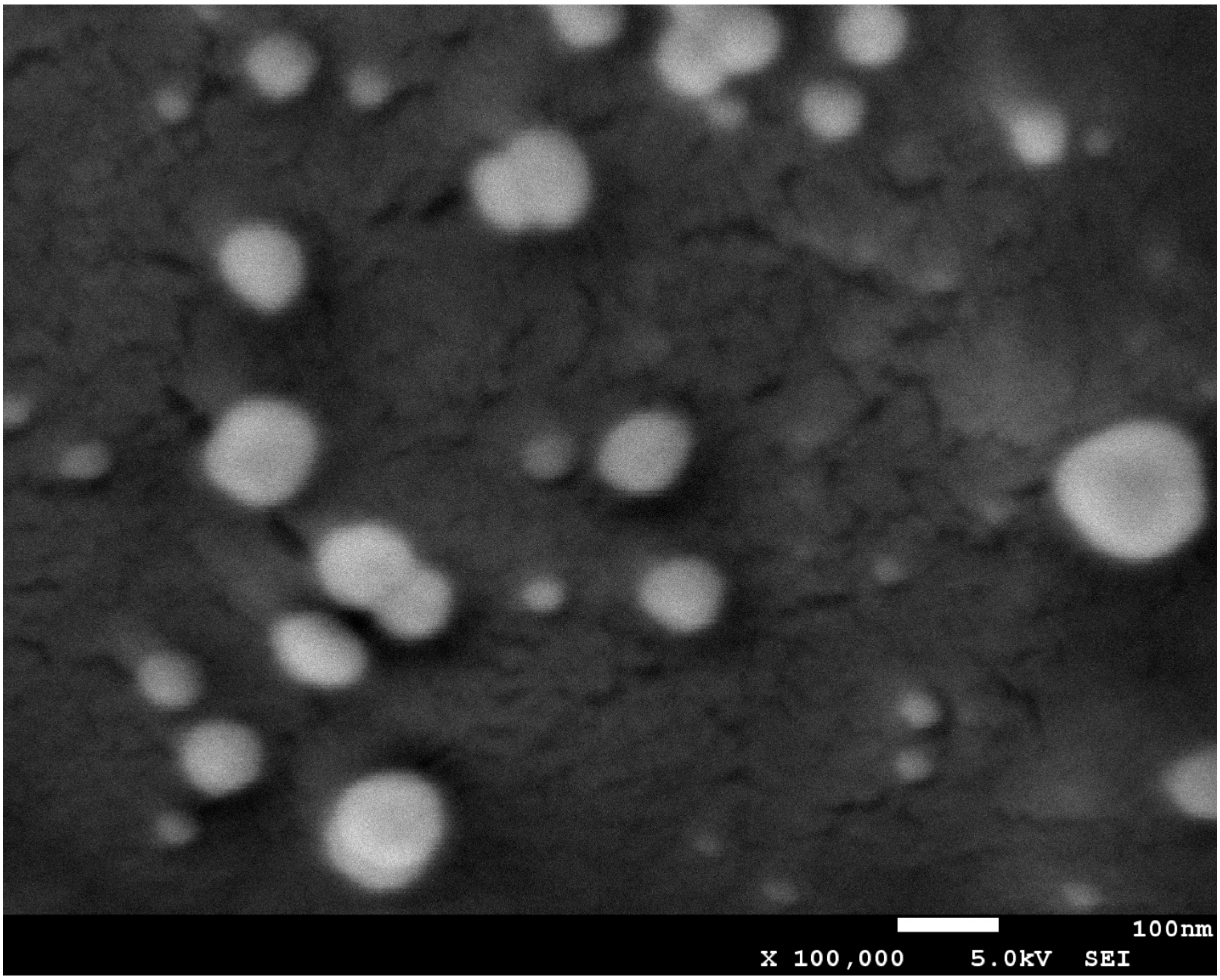

Figure S6. Full size of Cryo-SEM images of liposomal formulations with encapsulated fisetin, representation of Figure 8C.

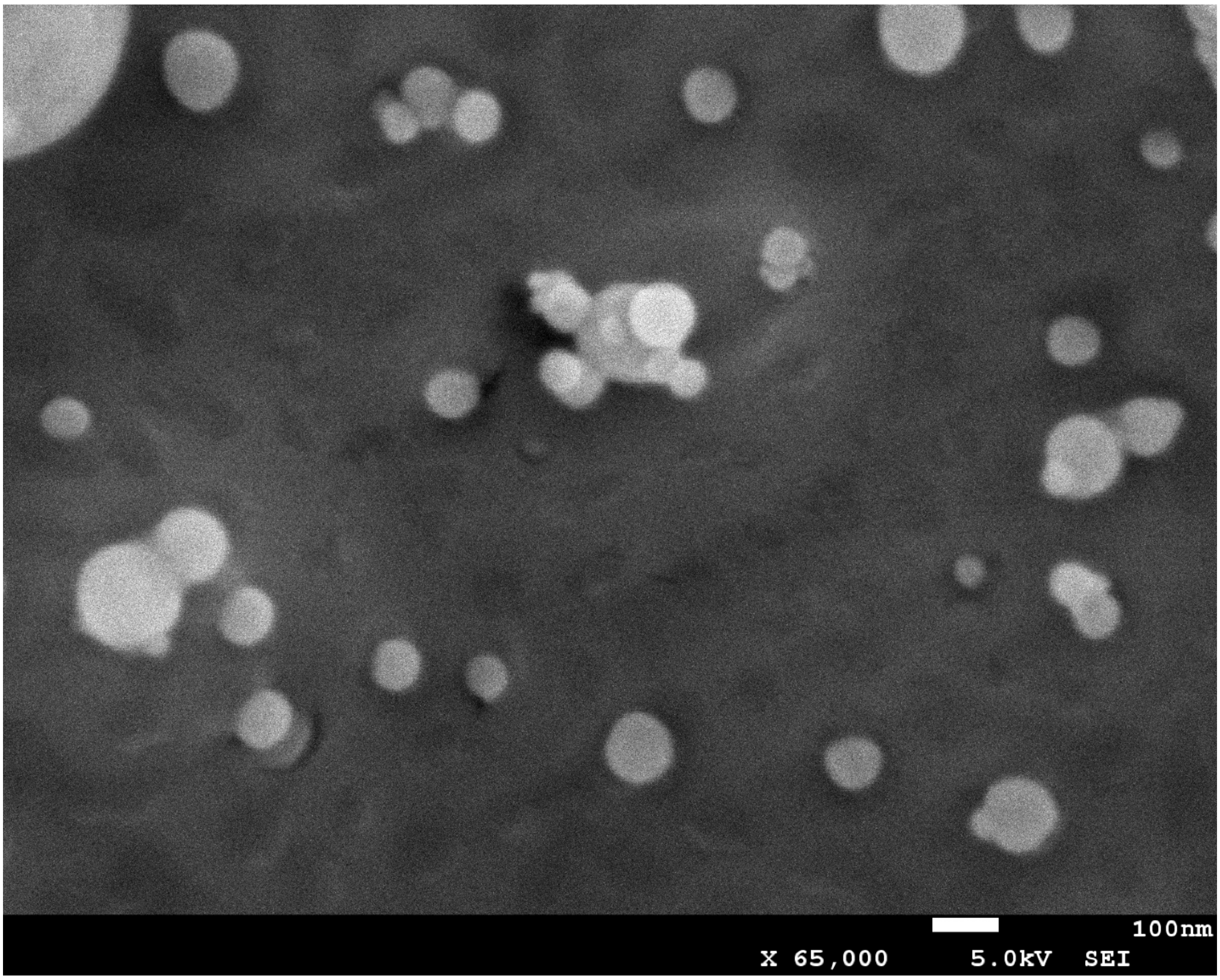

Figure S7. Full size of Cryo-SEM images of liposomal formulations with encapsulated fisetin, representation of Figure 8D.

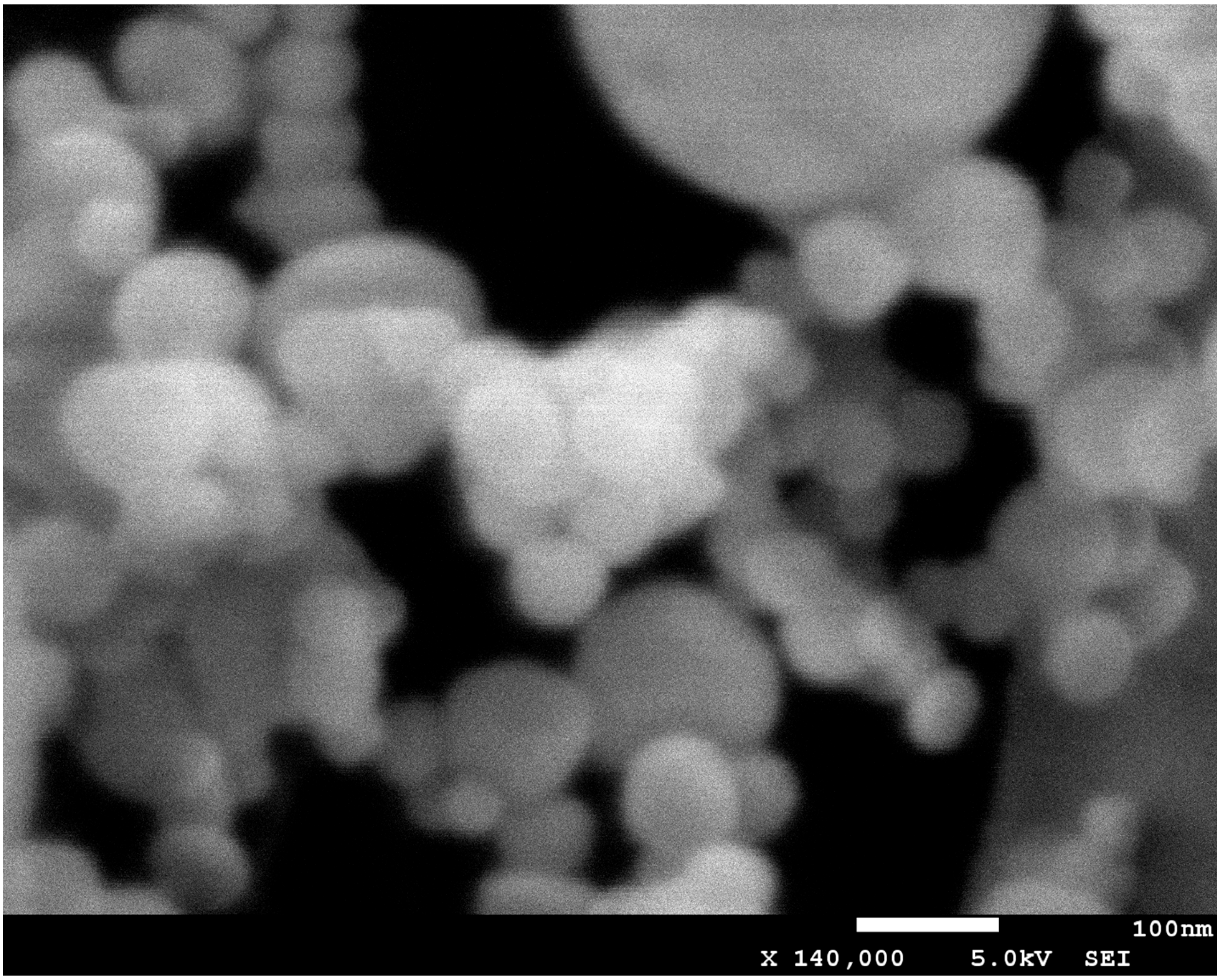

Figure S8. Full size of Cryo-SEM images of chain-like structures formed by fisetin-encapsulated liposomes, representation of Figure 8E.

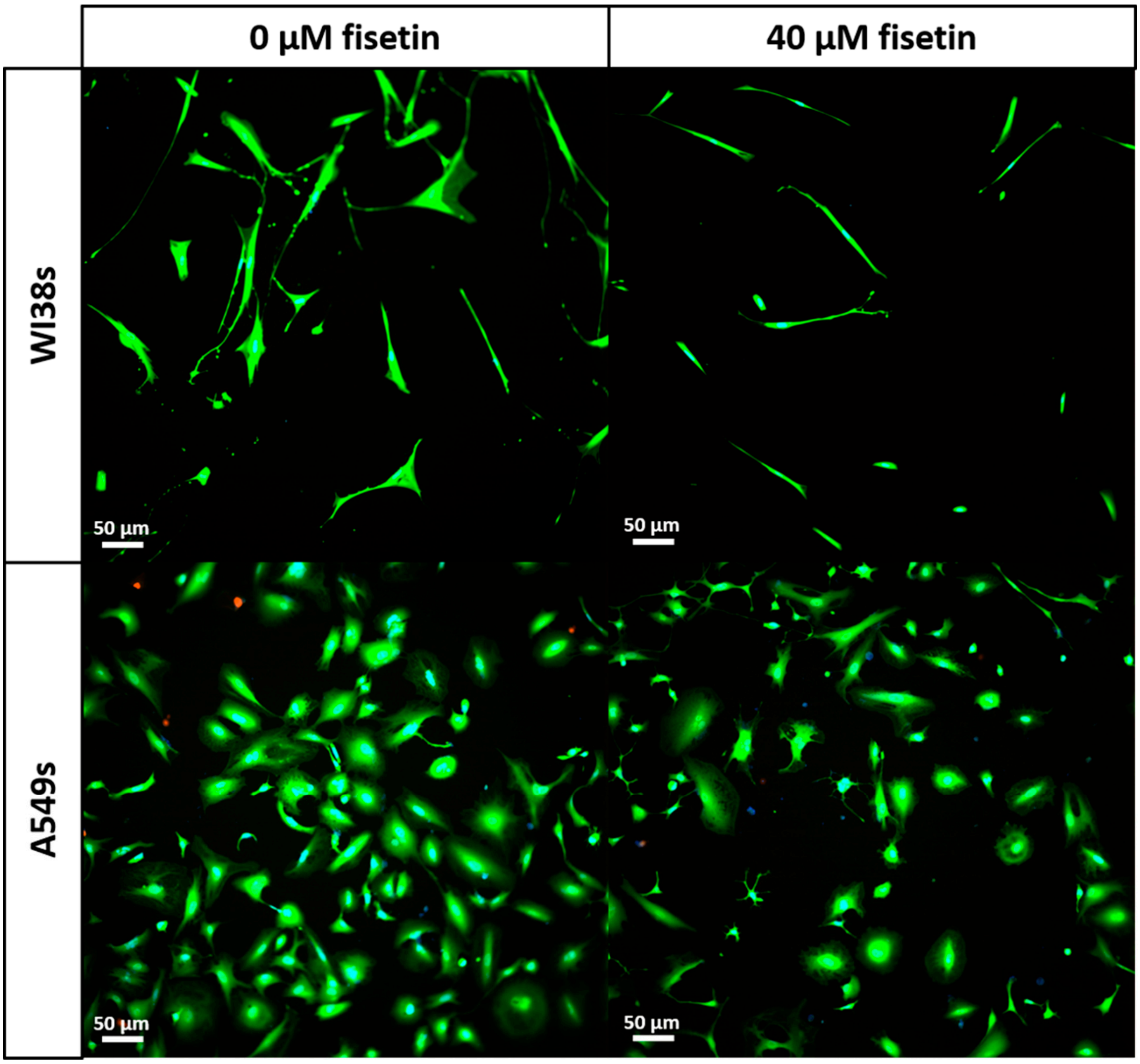

Figure S9. Representative images of morphological changes observed in senescent cells during live/dead viability assay after treatment with fisetin. s – senescent.

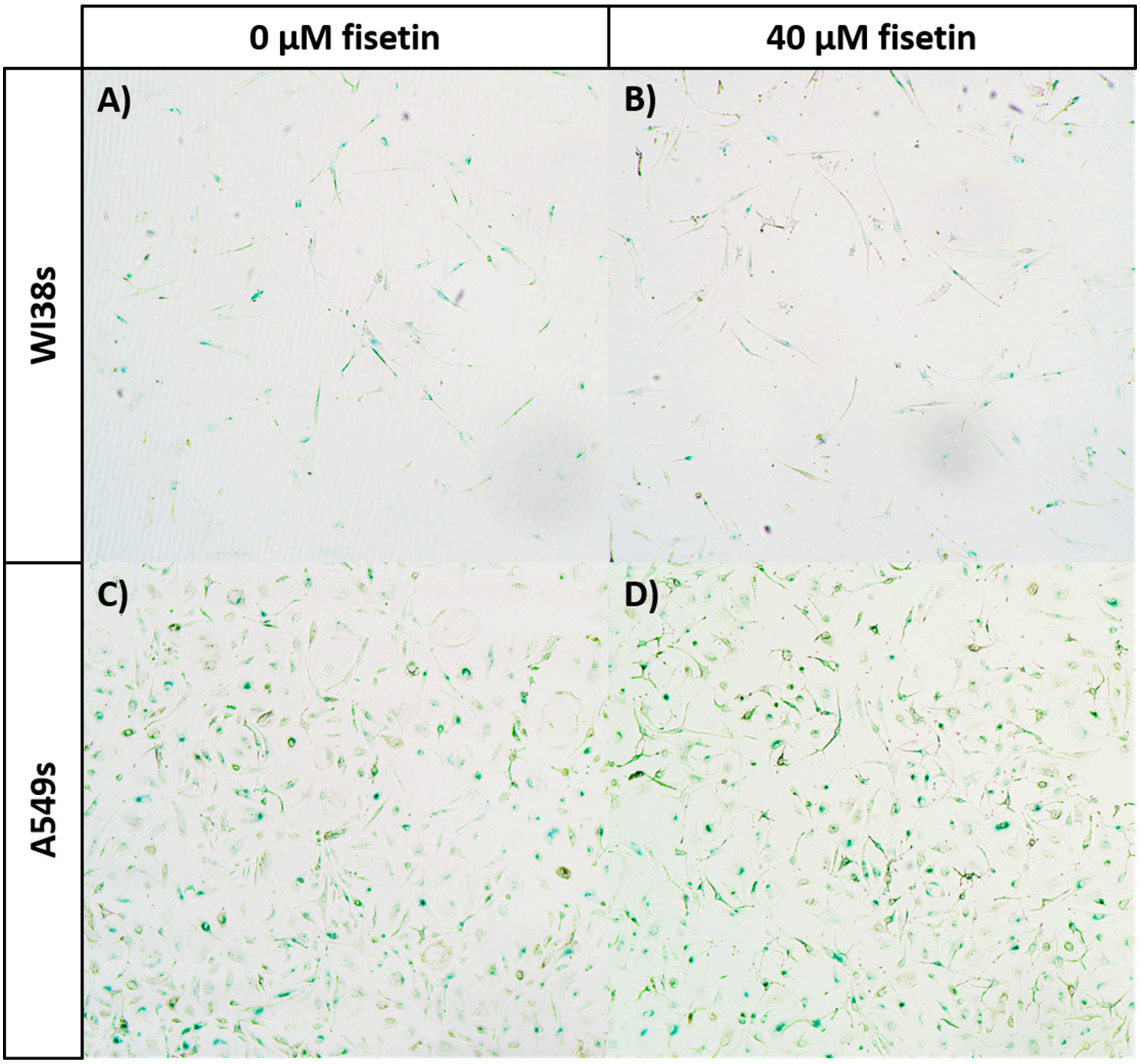

Figure S10. Representative images of SA-β-gal staining of senescent cells after treatment with fisetin taken on inverted light microscope. A) Control of senescent WI38 and B) senescent WI38 after incubation with fisetin for 48 hours. C) Control of senescent A549 after incubation with fisetin for 48 hours. Magnification x40. s – senescent.

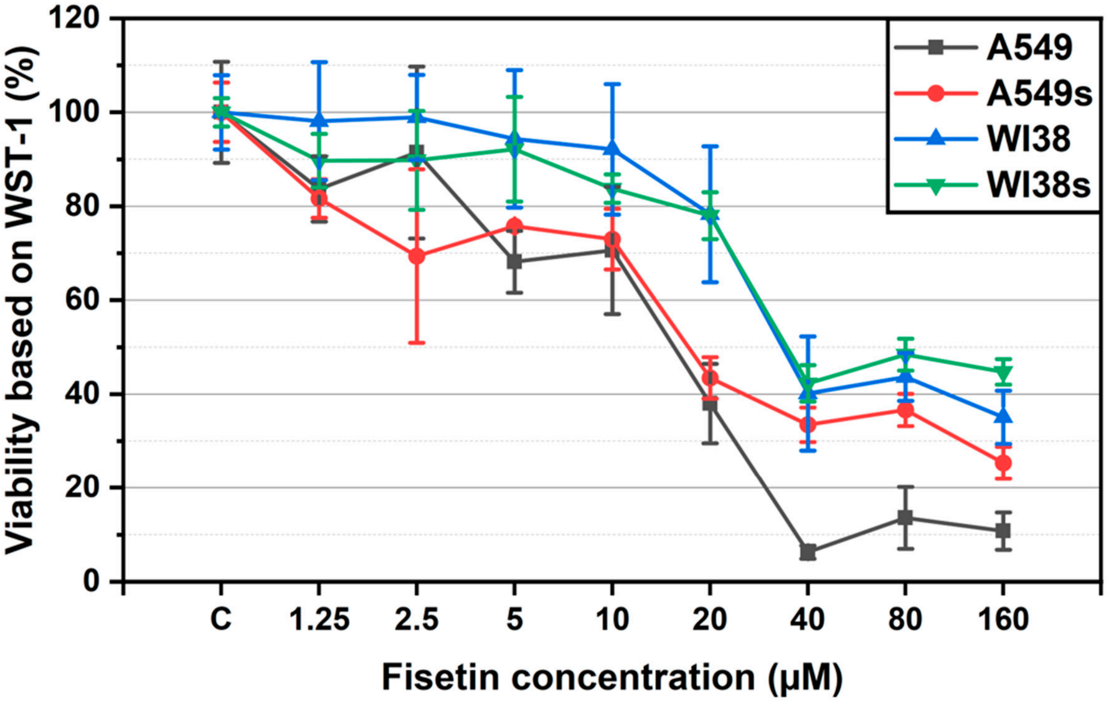

Figure S11. WST-1 assay results after 48-hour treatment with varying concentrations of fisetin. This assay assesses cell viability based on metabolic activity. The results show a concentration-dependent decrease in metabolic activity, however, other experiments indicate that cell viability is not significantly affected.
